# Role of Heat Shock Proteases in Quorum-Sensing-Mediated Regulation of Biofilm Formation by *Vibrio* Species

**DOI:** 10.1128/mBio.02086-17

**Published:** 2018-01-02

**Authors:** Kyung-Jo Lee, You-Chul Jung, Soon-Jung Park, Kyu-Ho Lee

**Affiliations:** a Department of Life Science, Sogang University, Seoul, South Korea; b Department of Environmental Medical Biology and Institute of Tropical Medicine, Yonsei University College of Medicine, Seoul, South Korea; University of California, Santa Cruz; University of Washington

**Keywords:** ClpPA, Lon, quorum-sensing master regulators, *Vibrio* species

## Abstract

Capsular polysaccharide (CPS) is essential for the dispersal of biofilms formed by the pathogenic bacterium *Vibrio vulnificus*. CPS production is induced by the quorum-sensing (QS) master regulator SmcR when biofilms mature. However, *V. vulnificus* biofilms formed under heat shock conditions did not exhibit the dispersion stage. Transcripts of the CPS gene cluster were at basal levels in the heat-exposed cell owing to reduced cellular levels of SmcR. At least two proteases induced by heat shock, ClpPA and Lon, were responsible for determining the instability of SmcR. *In vitro* and *in vivo* assays demonstrated that SmcR levels were regulated via proteolysis by these proteases, with preferential proteolysis of monomeric SmcR. Thus, CPS production was not induced by QS when bacteria were heat treated. Further studies performed with other *Vibrio* species demonstrated that high temperature deactivated the QS circuits by increased proteolysis of their QS master regulators, thus resulting in alterations to the QS-regulated phenotypes, including biofilm formation.

## IMPORTANCE

The term "quorum-sensing mechanism" is used to describe diverse bacterial cell density-dependent activities that are achieved by sensing of the signaling molecules and subsequent signal transduction to the master regulators. These well-known bacterial regulatory systems regulate the expression of diverse virulence factors and the construction of biofilms in pathogenic bacteria. There have been numerous studies designed to control bacterial quorum sensing by using small molecules to antagonize the quorum-sensing regulatory components or to interfere with the signaling molecules. In the present study, we showed that the quorum-sensing regulatory circuits of pathogenic *Vibrio* species were deactivated by heat shock treatment via highly increased proteolysis of the master transcription factors. Our results showed a new mode of quorum deactivation which can be achieved under conditions of high but nonlethal temperature even if the ambient signaling molecules may reach the levels representing high cell density.

## INTRODUCTION

Biofilms provide advantages for the survival of pathogens against a variety of stresses, including host defense, resulting in increased potential for pathogenicity in host environments. In cases of *Vibrio vulnificus* infection causing primary septicemia and gastroenteritis in humans (1, 2), mutant strains that were unable to form mature biofilms had severely impaired virulence in a mouse model (3, 4). Polysaccharides in the extracellular polymeric matrix (EPM) of *V. vulnificus* biofilms, which are produced in a stage-specific manner during the biofilm formation process, are critical for the successful completion of a stage or for the transition to the next stage. Thus, the ability of *V. vulnificus* to form biofilms is accompanied by the timely production of polysaccharides, such as a lipopolysaccharide (LPS; 5) at the initial stage, at least three kinds of exopolysaccharides (EPS; 3) during the maturation stage, and a capsular polysaccharide (CPS; 6) at the final dispersal stage. *V. vulnificus* CPS facilitates the dispersal of bacterial cells from biofilm structures after the maturation stage since it provides the hydrophilic basis of biofilm EPM due to the presence of a negatively charged sugar. Its production is regulated by quorum sensing (QS) via the induction of the CPS gene cluster transcription by QS master regulator SmcR (6).

The term “bacterial QS” is used to describe diverse cell density-dependent activities that are achieved via the accumulation of signaling molecules called autoinducers (AIs) (7). In addition to well-known AIs such as acylated homoserine lactones and (2S,4S)-2-methyl-2,3,3,4-tetrahydroxytetrahydrofuran borate (AI-2), (S)-3-hydroxytridecan-4-one and 3,5-dimethylpyrazin-2-ol have been recently identified in the pathogenic *Vibrio* spp. (8, 9). Once extracellular AIs are recognized by membrane-bound proteins, their signals are further transduced to the transcription factors called master QS regulators. Those include *V. harveyi* LuxR, *V. parahaemolyticus* OpaR, *V. cholerae* HapR, and *V. vulnificus* SmcR, which regulate the expression of the representative target genes responsible for bioluminescence (10), colony opacity (11), extracellular HA/protease (12), and starvation-induced maintenance of culturability (13), respectively.

Among the diverse phenotypes controlled by QS in *V. vulnificus*, the sizes of mature biofilms have been reported to be negatively regulated by QS (4, 6). Its master QS regulator, SmcR, activates the transcription of genes encoding the proteins required for biofilm dispersal such as the negatively charged capsular polysaccharide (CPS; 6) and exoproteolytic elastase (VvpE; 4). It is speculated that QS signals reach the threshold concentration in the mature biofilms to induce QS for the next dispersal stage of the biofilm formation process (6). The dispersal of bacterial cells from the biofilm structures is believed to be critical in the cycling of biofilm-plankton life styles, the colonization of other available surface habitats, and the survival of remaining cells in appropriately sized biofilms. QS is also important in biofilm formation by other *Vibrio* species by regulating expression of factors critical for the specific stages of biofilm formation, such as the CPS of *V. parahaemolyticus*, EPS of *V. cholerae*, and flagella of *V. harveyi* (14–16).

From the screening assays used to identify the environmental parameters influencing biofilm formation, we have identified high but nonlethal temperatures inducing the formation of bigger biofilms by *V. vulnificus*. Subsequent investigations showed that this was mainly caused by reduced production of CPS due to reduced cellular levels of SmcR. In this study, we elucidated the regulatory mechanisms to determine the cellular contents of *V. vulnificus* SmcR via the involvement of well-known heat shock proteases. Furthermore, this report presents evidence demonstrating that the same types of heat shock regulation for QS are conserved in other *Vibrio* species.

## RESULTS

### Increased biofilm formation by *V. vulnificus* under heat shock conditions.

**FIG 1:**
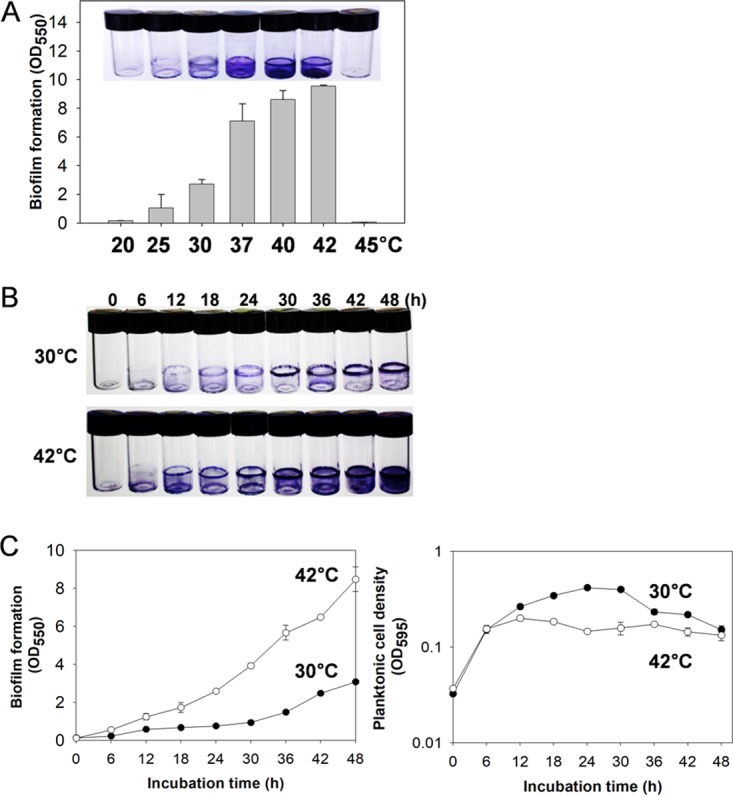
Biofilm formation by *V. vulnificus* under heat shock conditions. (A) Biofilm formation at various incubation temperatures. Wild-type *V. vulnificus* was inoculated into the AB-fumarate medium and incubated for 48 h at 20, 25, 30, 37, 40, 42, and 45°C. Biofilms formed on the borosilicate surface were observed by staining with crystal violet. Crystal violet dye associated with biofilms was eluted with ethanol, and the amounts of dye were measured by spectrophotometry at 550 nm. (B) Time series for the formation of biofilms in borosilicate tubes. Wild-type *V. vulnificus* was incubated in the AB-fumarate medium for 48 h at 30°C (upper panel) and 42°C (lower panel). Formed biofilms at the designated time points were observed by staining with crystal violet. (C) Determination of crystal violet-stained biofilms and planktonic biomasses. Stained crystal violet in biofilms was measured by spectrophotometry at 550 nm (left panel). The planktonic bacterial biomass in the same assay tube was estimated by spectrophotometry at 595 nm (right panel).

To examine the effect of incubation temperature on biofilm formation by *V. vulnificus*, wild-type (WT) strain MO6-24/O was incubated for 48 h under conditions of various temperatures ranging from 20°C to 45°C. Biofilms formed on borosilicate tubes gradually increased in size as the incubation temperature increased to 42°C (Fig. 1A). However, no apparent biofilm formation was observed in the 45°C incubation, in which bacterial growth was almost negligible (see Fig. S1 in the supplemental material). This marine bacterium, when entering humans, would be exposed to high temperatures in the gut environments whose maximum may reach above 40°C (17). *V. vulnificus* formed the biggest biofilms at 42°C without showing heat-mediated attenuation of bacterial growth (Fig. S1). Therefore, further experiments designed to reveal the effects of high temperature on *V. vulnificus* were performed by incubating cells at 42°C. Levels of *V. vulnificus* biofilms formed on borosilicate tubes at 42°C were highly increased during the whole incubation period, at least up to 48 h, compared to the biofilms formed at 30°C (Fig. 1B). In contrast, the planktonic cell masses in the biofilm assay tubes, which were monitored by spectrometry at 595 nm, were slightly decreased at 42°C during the maturation stage of biofilms (Fig. 1C right panel) compared to the planktonic cell masses seen at 30°C. The estimated amounts of the dye staining the biofilms at 42°C were 4 to 5 times greater than those seen with the biofilms at 30°C (Fig. 1C left panel).

10.1128/mBio.02086-17.1 FIG S1  Growth curves of *V. vulnificus*. Wild-type *V. vulnificus* was inoculated into AB-fumarate medium and cultured for 8 h in shaking incubators (200 rpm) at 30, 37, 42, and 45°C. Every 30 min, planktonic bacterial biomass was measured by spectrophotometry at 595 nm (OD_595_). Download FIG S1, TIF file, 0.03 MB. Copyright © 2018 Lee et al. 2018 Lee et al. This content is distributed under the terms of the Creative Commons Attribution 4.0 International license.

### Decreased CPS production by *V. vulnificus* cells grown under heat shock conditions.

**FIG 2:**
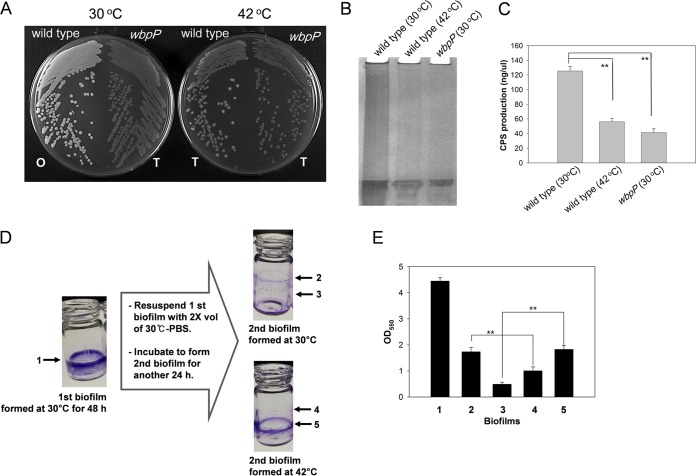
Capsular polysaccharide (CPS) production by *V. vulnificus* under heat shock conditions. (A) Colony morphology. Wild-type and *wbpP* mutant strains of *V. vulnificus* were grown on LBS agar plates for 12 h at 30°C and 42°C, and then the opacity of the bacterial colonies was examined. "O" and "T" indicate the colonies with opaque and transparent morphologies, respectively. (B) Polyacrylamide gel electrophoresis of the CPS extracts. CPS preparations extracted from each strain grown at 30°C and 42°C for 48 h were analyzed by electrophoresis in a 5% stacking polyacrylamide gel and subsequent staining with Stains-All. (C) Quantification of the CPS contents. Concentrations of the extracted CPS were quantified using d-galacturonic acid as a standard as described by Taylor (53). The estimated concentrations were expressed as nanograms of galacturonic acid equivalents in 1 μl of CPS extract. (D and E) Dispersal of biofilms. (D) Biofilms formed by wild-type *V. vulnificus* in 2 ml AB-fumarate broth at 30°C (labeled "1") were further incubated in 4 ml PBS at 30°C or 42°C to allow the second biofilms (labeled "2" and "4") to be formed by the cells dispersed from the first biofilms. The remaining first biofilms (labeled "3" and "5") and newly formed second biofilms around the air-liquid interfaces were stained with crystal violet. (E) The amounts of crystal violet were estimated by spectrophotometry at 550 nm.

We have previously reported that a *V. vulnificus* mutant defective in the production of CPS (i.e., the *wbpP* mutant) showed increased biofilm formation compared to the wild type (6). To confirm whether the CPS synthesis of *V. vulnificus* depends on the incubation temperature, the colony opacity of *V. vulnificus* grown at 30°C or 42°C on an AB-fumarate plate was observed. The CPS-negative *wbpP* mutant exhibited a translucent colony type, whereas the wild type grown at 30°C exhibited an opaque colony morphology. The wild-type *V. vulnificus* strain grown at 42°C, however, exhibited a transparent colony morphology (Fig. 2A). To confirm whether the translucent appearance of the *V. vulnificus* colonies formed at 42°C was due to a defect in CPS synthesis, CPS fractions were extracted from *V. vulnificus* grown on AB-fumarate agar plates at 30°C or 42°C for 48 h and were separated on a 5% stacking polyacrylamide gel. Stained CPS fractions from *V. vulnificus* grown at 42°C revealed basal levels, which was similar to the results seen with the *wbpP* mutant (Fig. 2B). The concentrations of CPS from the wild-type strain and the *wbpP* mutant grown at 30°C were 125 ± 6.3 and 41 ± 5.2 ng of galacturonic acid equivalents per μl of CPS preparation, respectively. The CPS concentration in the fraction extracted from the wild type grown at 42°C was 56 ± 4.2 ng of galacturonic acid equivalents per μl of CPS preparation (Fig. 2C). The increased sizes of biofilms formed by CPS-deficient mutants were speculated to be caused by reduced dispersion of cells from the mature biofilms due to the increased adherence and aggregation of the cells as previously suggested (6). To examine the effect of incubation temperature on the degree of dispersal, biofilms formed at 30°C were resuspended in 2× volumes of fresh phosphate-buffered saline (PBS; 137 mM NaCl, 2.7 mM KCl, 10 mM Na_2_HPO_4_, and 2 mM KH_2_PO_4_; pH 7.4) and further incubated at 30°C or 42°C for another 24 h (Fig. 2D and E). The first biofilms that had been formed at 30°C for 48 h (labeled "1" in Fig. 2D and E) were more highly retained at 42°C (labeled "5") than at 30°C (labeled "3"). In contrast, the second biofilms formed by the dispersed cells from the first biofilms were apparent at 30°C (labeled "2") but were minimal at 42°C (labeled "4").

### Downregulation of the CPS gene cluster due to significantly reduced levels of the transcription activator SmcR under heat shock conditions.

**FIG 3:**
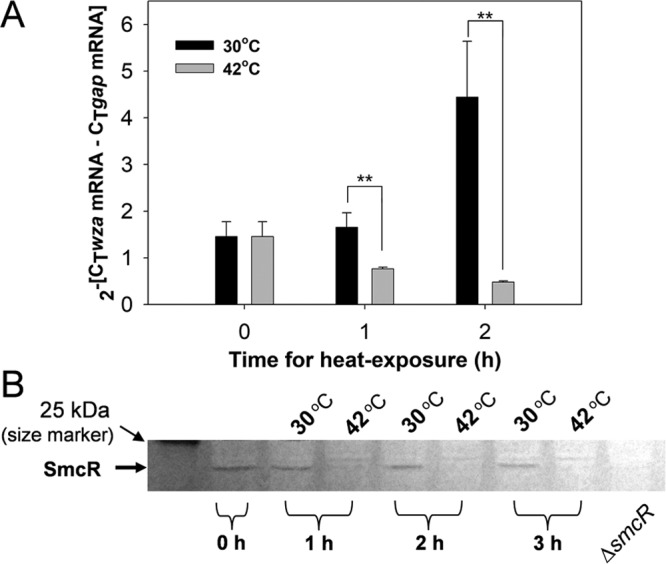
Transcription of the CPS gene cluster and cellular levels of SmcR under the heat shock conditions. (A) Cellular transcript levels of the CPS gene cluster. Wild-type *V. vulnificus* cells grown in the AB-fumarate medium to the late exponential phase (OD_595_ of 1.0) were divided and further incubated at 30°C and 42°C for 2 h. The transcript contents of the CPS gene cluster were determined by quantitative RT-PCR, and its expression levels were normalized to *gap* transcript levels. (B) Cellular levels of SmcR. Eighty micrograms of protein lysates of *V. vulnificus* cells which had been exposed to 30°C and 42°C for up to 3 h were fractionated by SDS-PAGE and then subjected to Western blot analysis using anti-SmcR polyclonal antibodies. As a negative control, Δ*smcR* mutant lysate was included in the same blot. The protein band corresponding to SmcR is indicated with an arrow.

In *V. vulnificus* strain MO6-24/O, CPS production is mainly dependent on the transcriptional regulation of the CPS biosynthesis gene cluster comprising at least 18 genes from *wza* to *wbfV* (CPS gene cluster; 6). To examine if the depleted production of CPS in heat-treated *V. vulnificus* was caused by a defect in its transcription, quantitative reverse transcriptase PCR (RT-PCR) was performed using the *wza* gene in the CPS gene cluster. Relative abundances of the transcript of the CPS gene cluster in *V. vulnificus* incubated at 30°C and 42°C were estimated by normalization to the transcripts of the glyceraldehyde-3-phosphate dehydrogenase (*gap*) gene in each sample. Compared to the *wza* transcript levels in cells grown at 30°C, the cellular levels of the *wza* transcript were significantly decreased in cells exposed to 42°C (Fig. 3A). Since transcription of the CPS gene cluster is activated by the QS master regulator SmcR, its contents in cells exposed to 42°C were examined. Western blot analysis using SmcR-specific antibodies showed that the levels of SmcR rapidly diminished in cells exposed at 42°C, and no apparent SmcR band was observed in samples incubated at 42°C for 1 h (Fig. 3B). In contrast, cells that were exposed to 30°C were well maintained for at least 3 h. These results led us to hypothesize the involvement of a heat shock protease(s) in the specific proteolysis of SmcR.

### Increased cellular levels of SmcR in the *clpP*, *clpA*, and *lon* mutants.

**FIG 4:**
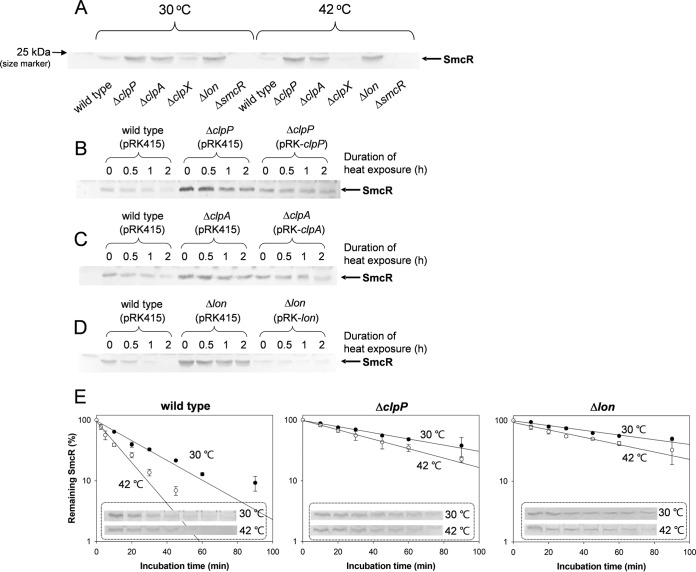
Cellular levels of SmcR in *V. vulnificus* mutant strains. (A) Cellular levels of SmcR in *V. vulnificus* mutant strains under the heat shock conditions. To identify the heat shock proteases involved in determining the cellular levels of SmcR, Western blot analysis was performed using *V. vulnificus* mutants defective in *clpP*, *clpX*, *clpA*, or *lon*. Eighty micrograms of protein lysates of *V. vulnificus* cells exposed to 30°C and 42°C for 3 h was subjected to Western blot analysis as described for Fig. 3B. As a negative control, Δ*smcR* mutant lysate was included in the same blot. The protein band corresponding to SmcR is indicated with an arrow. (B to D) Effects of the *clpP*, *clpA*, and *lon* mutations on the cellular content of SmcR. *V. vulnificus* strains, including the wild type carrying pRKA415, a Δ*clpP* mutant carrying pRK415, a Δ*clpP* mutant carrying pRK415-*clpP*, a Δ*clpA* mutant carrying pRK415, a Δ*clpA* mutant carrying pRK415-*clpA*, a Δ*lon* mutant carrying pRK415, and a Δ*lon* mutant carrying pRK415-*lon*, all of which had been exposed to 42°C for up to 2 h, were subjected to Western blot analysis using anti-SmcR antibodies. The protein bands corresponding to SmcR in mutants Δ*clpP* (B), Δ*clpA* (C), and Δ*lon* (D) are indicated with an arrow. (E) Endogenous degradation of SmcR in *V. vulnificus* strains. To determine the stability of SmcR in wild-type, Δ*clpP*, and Δ*lon V. vulnificus*, each strain was subjected to antibiotic chase assays as described in Materials and Methods. *V. vulnificus* cells exposed to 30°C or 42°C for various durations (up to 1.5 h) were harvested, and 80 μg of protein lysates was subjected to Western blot analysis using anti-SmcR polyclonal antibodies. The level of SmcR protein remaining in each strain is expressed as the percentage of SmcR intensity at time 0 and plotted against incubation time.

To identify the factor(s) responsible for the lowered levels of SmcR at high temperatures, the cellular contents of SmcR in the heat shock protease mutants of *V. vulnificus* were examined. Wild-type, Δ*clpP*, Δ*clpA*, Δ*clpX*, and Δ*lon* strains were grown at 30°C or 42°C, and their cell lysates were subjected to Western blotting with the SmcR-specific antibodies. Resultant blots showed that the intensities of the SmcR bands were apparently higher in the lysates of the Δ*clpP*, Δ*clpA*, and Δ*lon* strains than in those of the wild-type strain grown at 30°C. Moreover, the increased levels of SmcR in these mutants were maintained even at the higher incubation temperature, while the SmcR band was not detected in wild-type cells exposed to 42°C as was shown in Fig. 3B (Fig. 4A). To confirm that *V. vulnificus* ClpP, ClpA, and Lon are heat shock proteins, the effects of heat shock on cellular levels of ClpP, ClpA, and Lon were observed using anti-ClpP, anti-ClpA, and anti-Lon polyclonal antibodies. As expected, these proteins were apparently present in cells grown at the normal incubation temperature, i.e., at 30°C, and their cellular levels were induced under the heat shock conditions, i.e., at 42°C (Fig. S2).

10.1128/mBio.02086-17.2 FIG S2  Western blot analyses of ClpP (A), ClpA (B), and Lon (C). Crude lysates of wild-type and mutant *V. vulnificus* cells grown at 30°C or 42°C were used to compare the cellular contents of ClpP, ClpA, and Lon. Each protein appeared as an immunoreactive band as indicated with an arrow. Lane 1, a protein size marker; lane 2, wild type grown at 30°C; lane 3, wild type grown at 42°C; lane 4, negative controls (Δ*clpP*, Δ*clpA*, or Δ*lon* mutant). Download FIG S2, TIF file, 0.2 MB. Copyright © 2018 Lee et al. 2018 Lee et al. This content is distributed under the terms of the Creative Commons Attribution 4.0 International license.

### Effects of mutations in *clpP*, *clpA*, and *lon* on SmcR contents.

To examine the effects of *clpP*, *clpA*, and *lon* mutations on the cellular levels of SmcR, the wild-type strain harboring broad-host-range vector pRK415, the mutant (Δ*clpP*, Δ*clpA*, and Δ*lon*) strains harboring pRK415, and the mutant strains complemented with the corresponding intact genes in pRK415 (e.g., pRK-*clpP*, pRK-*clpA*, or pRK-*lon*) were exposed to 42°C for up to 2 h (Fig. 4B to D). Heat-exposed cells were subjected to Western blot analysis using anti-SmcR antibodies, and the resultant SmcR band intensities were compared with those of the wild type. The cellular content of SmcR was gradually decreased in wild-type cells as the duration of exposure to 42°C increased at least up to 2 h. In contrast, much higher cellular SmcR levels were apparent in the three mutant strains than in the wild-type strain exposed for the same incubation time at 42°C. The three mutant strains were also relatively less sensitive to the exposure time at 42°C than the wild-type strain. However, Δ*clpP* and Δ*clpA* mutants showed cellular levels of SmcR similar to those seen with the wild type when they were complemented with plasmids pRK-*clpP* and pRK-*clpA*, respectively (Fig. 4B and C). In the case of the Δ*lon* mutant harboring pRK-*lon*, SmcR was rarely detected even in the cells that had not been exposed to 42°C (Fig. 4D).

These results suggest that the sensitivity of SmcR to the ClpAP and Lon proteases plays a role in the decreased cellular levels of SmcR in heat-exposed cells. To further investigate this phenomenon, we compared data corresponding to the half-life of SmcR in *V. vulnificus* cells grown at 30°C and 42°C, using antibiotic chase experiments as described in Materials and Methods. In the wild-type strain incubated at 30°C, SmcR had a half-life of 17.7 (+1.1) min, but SmcR showed a highly increased half-life of 58.7 (+8.7) min or 76.0 (+2.6) min in the Δ*clpP* strain or the Δ*lon* strain incubated at 30°C, respectively (Fig. 4E). When bacterial cells were incubated at 42°C, the estimated half-lives of SmcR in the wild-type, Δ*clpP*, and Δ*lon* strains were 6.2 (+2.5), 38.1 (+6.6), and 45.0 (+6.5) min, respectively.

### Effects of mutations in *clpP*, *clpA*, and *lon* on biofilm formation and CPS production.

**FIG 5:**
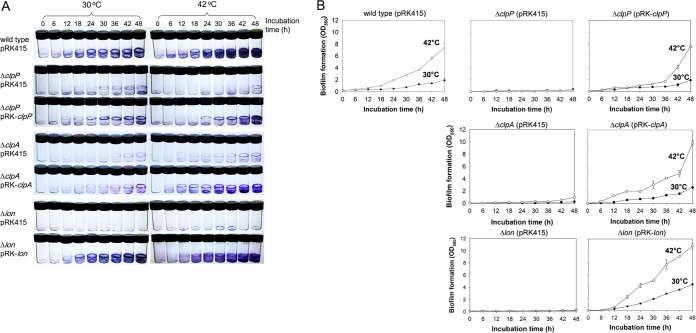
Effects of the *clpP*, *clpA*, and *lon* mutations on biofilm formation. *V. vulnificus* strains used as described for Fig. 4 were incubated in the AB-fumarate medium supplemented with 3 μg/ml tetracycline to form biofilms at 30°C (left panels in panel A; closed circles in panel B) and 42°C (right panels in panel A; open circles in panel B). The biofilms formed for the designated incubation times were stained with crystal violet (A), and the levels of the stained dyes were estimated as described for Fig. 1 (B).

The abilities of the Δ*clpP*, Δ*clpA*, and Δ*lon* mutants to form biofilms were observed at 30°C and 42°C. All three mutants produced significantly decreased biofilms at 30°C compared to the wild type or to each mutant complemented with the corresponding genes (Fig. 5A). In addition, these mutants did not show heat shock-responsive characteristics with respect to biofilm formation, such as the increased ability to form biofilms at 42°C shown by the wild type. Both the Δ*clpP* and the Δ*clpA* mutants formed approximately 5 to 7 times less biofilm than the wild-type cells at 30°C and approximately 8 to 37 times less biofilm than the wild-type cells at 42°C (Fig. 5B). These levels of biofilm formation by Δ*clpP* and Δ*clpA* mutants were restored to the wild-type level when the mutants were complemented with the intact genes. The Δ*lon* mutant almost entirely lost its ability to form biofilms at both 30°C and 42°C. It is noteworthy that the Δ*lon* mutant harboring pRK-*lon* formed biofilms at highly increased levels even at 30°C, and those biofilms were 2.3 times bigger than the biofilms formed by the wild type at 30°C. The planktonic cell masses of the Δ*clpP*, Δ*clpA*, and Δ*lon* mutant strains in biofilm assay tubes showed almost the same levels as those seen with the wild type (data not shown). Thus, these mutants did not show significantly attenuated growth in the medium used in this biofilm assay, and the decreases in formation of biofilms by the three mutants were not due to reduced growth.

**FIG 6:**
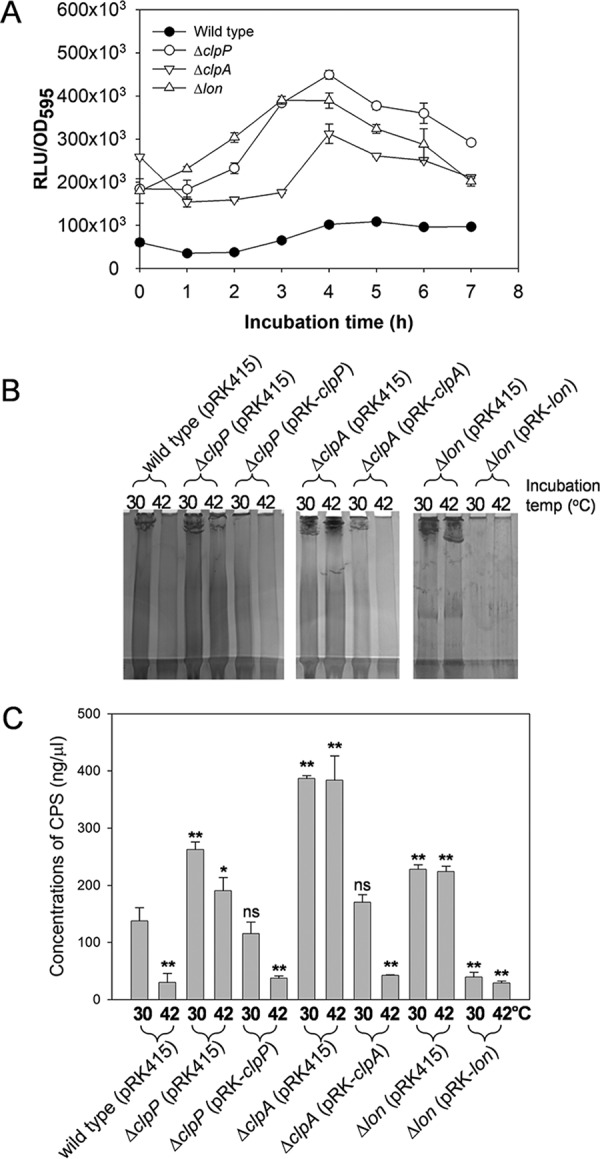
Effects of the *clpP*, *clpA*, and *lon* mutations on the expression of the CPS gene cluster and production of CPS. (A) Effects of the *clpP*, *clpA*, and *lon* mutations on the expression of the CPS gene cluster. Wild-type, Δ*clpP*, Δ*clpA*, and Δ*lon* strains carrying a *luxAB-*transcription reporter fusion for the CPS cluster (pCB014; 3) were incubated in the AB-fumarate medium supplemented with 3 μg/ml tetracycline. At the indicated time points, cell mass and light production levels were determined by using spectrophotometry (OD_595_) and luminometry (relative light units [RLU]), respectively. Specific luciferase activity is indicated by plotting the normalized value as RLU per OD_595_. (B and C) Effects of the *clpP*, *clpA*, and *lon* mutations on CPS production. (B) The *V. vulnificus* strains used as described for Fig. 4 were grown at 30°C or 42°C, and their CPSs were fractionated. CPS extracts were then visualized in polyacrylamide gels and quantified as described for Fig. 2. (C) The estimated carbohydrate concentrations are expressed in nanograms of galacturonic acid equivalents in 1 μl of CPS extract.

To examine if the transcription of the CPS gene cluster was affected by the *clpP*, *clpA*, or *lon* mutations due to increased cellular levels of SmcR in the three kinds of heat shock protease mutants, the promoter activity of a plasmid with a *luxAB*-transcription reporter fusion, including the promoter of the cluster and its upstream region (pCB014; 3), was monitored in the wild-type and mutant strains (Fig. 6A). Expression levels of pCB014 in all three mutants increased by approximately 3-fold to 4-fold compared to the same fusion in the wild type during the whole incubation periods. Since it was previously found that CPS production by *V. vulnificus* was regulated mainly at the transcription level and that SmcR is an activator for the transcription of the CPS gene cluster (6), the amounts of CPS produced by the wild-type and mutant strains harboring plasmids were compared (Fig. 6B). The wild-type cells produced 4.6-fold less CPS at 42°C than at 30°C. The estimated amounts of CPS were 138 ± 23 and 30 ± 16 ng of galacturonic acid equivalents in 1 μl of extract fractionated from wild-type cells grown at 30°C and 42°C, respectively (Fig. 6C). SDS-PAGE analyses of the CPS extracted from mutant cells showed that all three mutants grown at both 30°C and 42°C produced more CPS than the wild type grown at 30°C. The estimated amounts of CPS were 263 ± 13.2, 387 ± 5.0, and 228 ± 7.9 ng of galacturonic acid equivalents in 1 μl of extract fractionated from Δ*clpP*, Δ*clpA*, and Δ*lon* strains grown at 30°C, respectively (Fig. 6C). Complementation of the Δ*clpP* and Δ*clpA* mutants with the intact genes led to the production of CPS at 30°C or 42°C at levels similar to those seen with the wild-type cells grown at 30°C or 42°C, respectively. However, the Δ*lon* mutant complemented with *lon* always produced significantly smaller amounts of CPS regardless of the incubation temperature.

### Specific proteolysis of recombinant SmcR by ClpPA and Lon.

**FIG 7:**
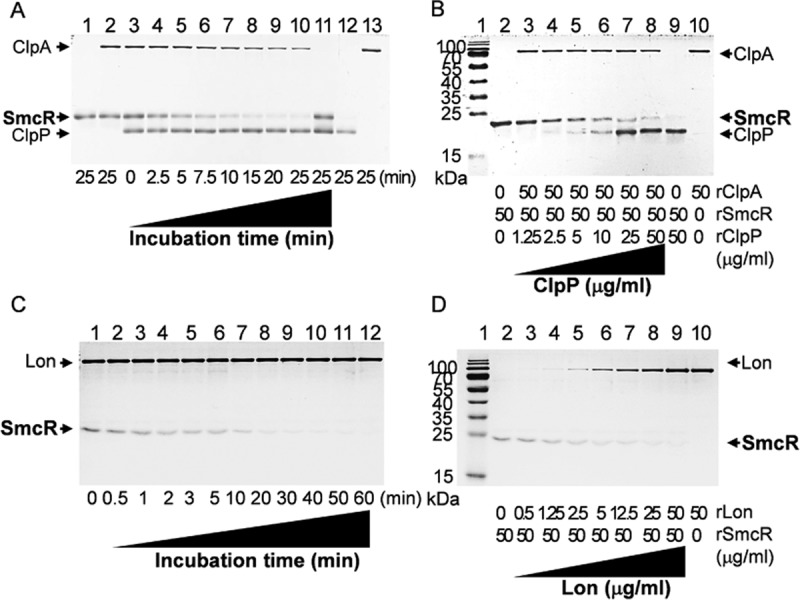
*In vitro* proteolysis of the SmcR by ClpPA and Lon. Data represent results of time series proteolysis of SmcR by ClpPA (A) and Lon (C). (A) rSmcR (2.0 μM; lane 1) was mixed with rClpP (2.2 μM each; lane 12) and rClpA (0.6 μM each; lane 13), and then the reaction mixtures were incubated for up to 25 min. For negative proteolytic controls of the ClpP/A reaction, rSmcR was incubated with rClpA (lane 2) or rClpP (lane 11) only. (C) Similarly, rSmcR was mixed with rLon (0.6 μM each) and incubated for up to 60 min. The resulting reaction mixtures were separated by SDS-PAGE and visualized by Coomassie blue staining. (B and D) Dose-dependent proteolysis by ClpPA (B) and Lon (D). Various concentrations of rClpP or rLon, as indicated in each lane, were added to a reaction mixture containing 2.0 μM rSmcR and incubated for 20 min (for rClpP) or 60 min (for rLon).

To elucidate whether SmcR is a substrate for the ClpPA- and Lon-driven proteolyses, *in vitro* proteolysis assays using recombinant proteins of SmcR (rSmcR), ClpP (rClpP), ClpA (rClpA), and Lon (rLon) were performed. Equal amounts of SmcR and ClpA were mixed in reaction buffer, and the proteolysis reaction was initiated by the addition of ClpP and stopped by boiling the mixture at the designated time points. Then, the amounts of added recombinant proteins were visualized by SDS-PAGE. ClpP did not cleave SmcR in the absence of ClpA (lane 11, Fig. 7A), and ClpP and A gradually cleaved SmcR in an incubation time-dependent manner (lanes 3 to 10, Fig. 7A); SmcR was almost completely cleaved in 20 min. In addition, ClpPA proteolysis of SmcR was dependent upon the added concentrations of ClpP (lanes 2 to 8, Fig. 7B). SmcR was added to reaction mixtures containing ClpPX instead of ClpPA. As expected from the Western blotting assay shown in Fig. 4A, SmcR was not cleaved by ClpPX at all (Fig. S3), suggesting the specificity of ClpPA with respect to SmcR. Similarly, the proteolytic activity of Lon toward SmcR was examined. As shown in the ClpPA proteolysis reactions, Lon activity showed incubation time-dependent (Fig. 7C) as well as protease concentration-dependent (Fig. 7D) patterns of proteolysis.

10.1128/mBio.02086-17.3 FIG S3  *In vitro* proteolysis of rSmcR in the presence of ClpPA or ClpPX. Various concentrations of rClpP (0, 0.55, 1.1, and 2.2 μM) were added to a reaction buffer containing 2.0 μM rSmcR and 0.6 μM ClpA (lanes 1 to 4) or 1.2 μM ClpX (lanes 5 to 8) and were incubated for 30 min. The resulting reaction mixtures were separated by SDS-PAGE and visualized by Coomassie blue staining. Download FIG S3, TIF file, 0.1 MB. Copyright © 2018 Lee et al. 2018 Lee et al. This content is distributed under the terms of the Creative Commons Attribution 4.0 International license.

### Differential sensitivity of the recombinant SmcR proteins to proteolysis.

**FIG 8:**
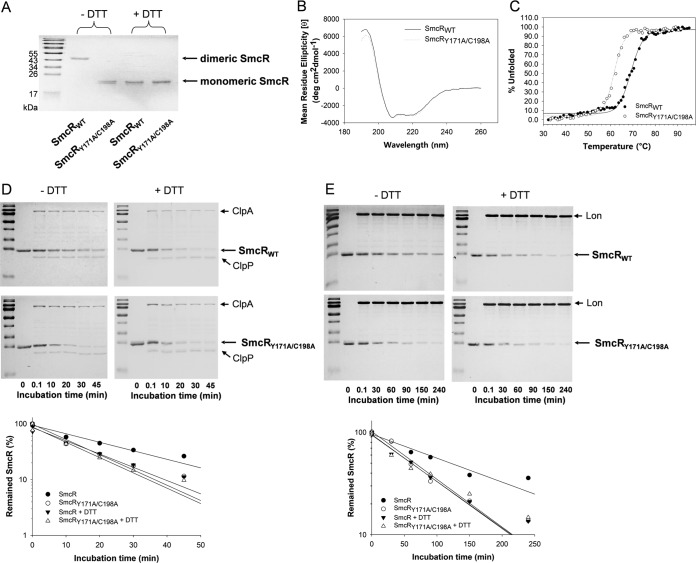
*In vitro* proteolysis of the monomeric and dimeric forms of SmcR by ClpPA and Lon. (A) Monomeric and dimeric forms of recombinant SmcR. The original SmcR (SmcR_WT_) and mutated SmcR (SmcR_Y171A/C198A_) were incubated in the presence (+DTT) or absence (−DTT) of 1 mM DTT and fractionated by SDS-PAGE. The protein bands were visualized by Coomassie blue staining. Monomeric and dimeric forms are designated with arrows. (B) Secondary structure assay of rSmcR. Changes in the secondary structure of SmcR_WT_ and SmcR_Y171A/C198A_ were monitored by scanning each recombinant protein (2.3 mg/ml) at 200 to 250 nm. The mean ellipticity of SmcR_WT_ and SmcR_Y171A/C198A_ was recorded and is indicated with solid and dotted lines, respectively. (C) Thermal stability assay of rSmcR. Thermal denaturation profiles of SmcR_WT_ and SmcR_Y171A/C198A_ (0.23 mg/ml each) were monitored by temperature-dependent CD. The change of ellipticity at 222 nm was used to calculate the percentages of unfolding protein, and the results are plotted with solid (SmcR_WT_) and dotted (SmcR_Y171A/C198A_) lines. (D) Time series proteolysis of monomeric and dimeric SmcR by ClpPA. SmcR_WT_ and SmcR_Y171A/C198A_ (2.0 μM each) were incubated with rClpP (2.2 μM) and rClpA (0.6 μM) in the absence (the left gels) or presence (the right gels) of 1 mM DTT. At various time points, the proteolysis reaction was stopped by adding a loading buffer and boiling. The resulting reaction mixtures were separated by SDS-PAGE and visualized by Coomassie blue staining. The levels of remaining rSmcR proteins were estimated by densitometric reading of the corresponding bands, and the relative levels of abundance compared with the rSmcR in the absence of ClpPA (lanes 2) were plotted against incubation time. (E) Time series proteolysis of monomeric and dimeric SmcR by Lon. SmcR_WT_ and SmcR_Y171A/C198A_ (2.0 μM each) were incubated with rLon (0.6 μM) in the absence (the left gels) or presence (the right gels) of 1 mM DTT. Proteolysis results are presented as described above.

SmcR has been shown to be a dimeric form when it binds to the regulatory region of a target gene (18), which is similar to the results seen with many transcription factors. To characterize the state of SmcR in determining the specific results of proteolysis by ClpPA and Lon under the heat shock conditions, a mutated SmcR protein was constructed. Tyr171 and Cys198, the amino acid residues in the C-terminal domain of SmcR, which have been reported to be important in the formation of dimeric SmcR (18), were substituted with alanine to produce SmcR_Y171A/C198A_. To examine the monomeric or dimeric states of the original (wild-type) SmcR (SmcR_WT_) and SmcR_Y171A/C198A_, the recombinant proteins were fractionated by SDS-PAGE in the presence or absence of a reducing agent, dithiothreitol (DTT). Under the nonreducing condition, most of the SmcR_WT_ was in the dimeric state, while SmcR_WT_ changed conformations to the monomeric state in the presence of DTT (Fig. 8A). In contrast, addition of DTT had no effect on SmcR_Y171A/C198A_, which always showed the monomeric state under both nonreducing and reducing conditions. Circular dichroism (CD) analysis was performed using both proteins to examine if mutations at Tyr171 and Cys198 of SmcR might have caused an alteration in the overall protein structure (Fig. 8B). SmcR_Y171A/C198A_ produced the same CD spectrum as SmcR_WT_, suggesting no change in the secondary structure of SmcR by substitution of 2 amino acid residues. However, the thermal unfolding assays performed by CD analysis of two proteins revealed that SmcR_Y171A/C198A_ was less stable than SmcR_WT_ (Fig. 8C). The melting temperatures of SmcR_WT_ and SmcR_Y171A/C198A_, which were calculated from the thermal unfolding curves, were 69.5°C and 62.1°C, respectively.

Proteolysis reactions using SmcR_WT_ in the presence or absence of DTT showed that the monomeric SmcR was more readily cleaved by ClpPA (Fig. 8D) and Lon (Fig. 8E) than the dimeric SmcR. In accordance with this observation, SmcR_Y171A/C198A_ was cleaved by ClpPA (Fig. 8D) and Lon (Fig. 8E) as readily as the monomeric SmcR_WT_. The estimated rates of proteolysis of SmcR by ClpPA were 88.0 and 59.1 nM/min for the monomeric and dimeric forms, respectively, and estimated rates of proteolysis of SmcR by Lon were 17.9 and 9.9 nM/min for the monomeric and dimeric forms, respectively (Fig. 8D and E).

### Regulation of QS by heat shock proteases in other *Vibrio* species.

**FIG 9:**
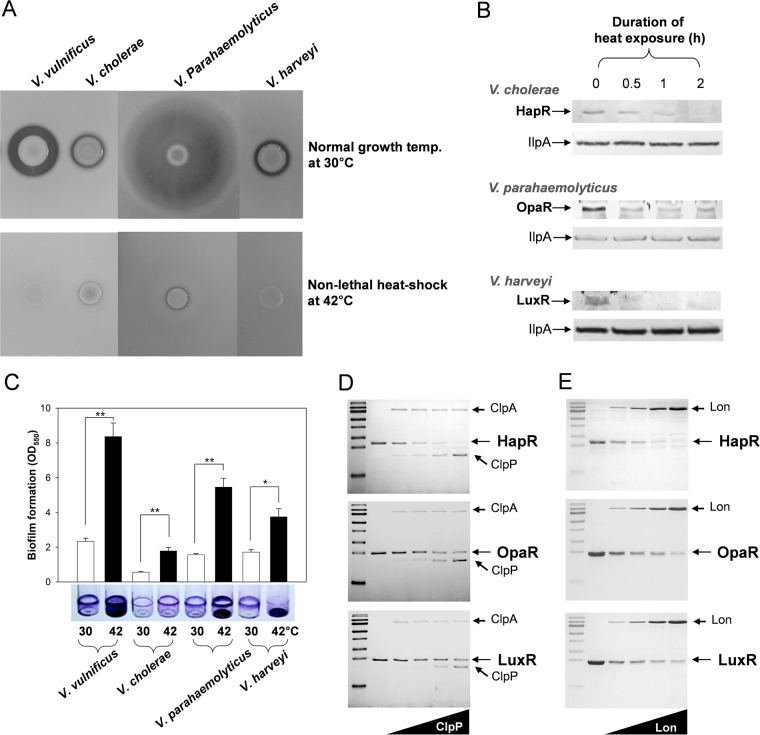
Effects of heat shock treatment on QS regulation in *Vibrio* species. (A) Exoproteolytic activities of *V. vulnificus*, *V. cholerae*, *V. parahaemolyticus*, and *V. harveyi*. To compare the degrees of QS regulation of various *Vibrio* species growing at normal temperature (30°C) and nonlethal high temperature (42°C), the total protease activities were monitored by spotting the cells on 2.5% skim milk agar plates. After 24 h of incubation at 30°C or 42°C, the cleared zones around the colonies were compared. (B) Cellular content of LuxR homologues of *Vibrio* species. *Vibrio* cells freshly grown at 30°C were exposed to 42°C for up to 2 h. Eighty micrograms of protein lysates of *Vibrio* cells were fractionated by SDS-PAGE and then subjected to Western blot analysis using anti-HapR, anti-OpaR, and anti-LuxR polyclonal antibodies. The IlpA content in the same samples was also monitored as a loading control, using anti-IlpA polyclonal antibodies (47). The protein bands corresponding to LuxR homologues and IlpA are indicated with arrows. (C) Biofilm formation by *Vibrio* species. *V. vulnificus*, *V. harveyi*, and *V. parahaemolyticus* cells inoculated into AB-fumarate medium and *V. cholerae* cells inoculated into LBS medium were incubated to form biofilm at 30°C and 42°C. Biofilms formed for 48 h were stained with crystal violet (the lower picture), and the levels of the stained dyes were estimated as described for Fig. 1. (D) *In vitro* proteolysis of LuxR homologues by ClpPA. Various concentrations of *V. vulnificus* rClpP, including 0 μM (lane 2), 0.3 μM (lane 3), 0.6 μM (lane 4), 1.1 μM (lane 5), and 2.2 μM (lane 6), were added to a reaction mixture containing 0.6 μM rClpA supplemented with 2.0 μM rHapR, rOpaR, or rLuxR and were then incubated for 60 min. The resulting mixtures were separated by SDS-PAGE and visualized by Coomassie blue staining. (E) *In vitro* proteolysis of LuxR homologues by Lon. Various concentrations of rLon of *V. vulnificus*, including 0 μM (lane 2), 0.08 μM (lane 3), 0.15 μM (lane 4), 0.3 μM (lane 5), and 0.6 μM (lane 6), were added to a reaction mixture containing 2.0 μM rHapR, rOpaR, or rLuxR and incubated for 60 min.

As described above, the *V. vulnificus* QS regulation could be turned off by a heat shock treatment. To investigate whether the heat shock also controls QS in other *Vibrio* species, the extracellular proteases were compared by inoculating those cells on skim milk agar plates and incubating at 30°C or 42°C. Expression of exoproteases was dependent upon QS in *V. vulnificus*, *V. cholerae*, and *V. harveyi* (12, 19, 20), and it is presumed to be similarly regulated in *V. parahaemolyticus* as well. The proteolytic activity of *Vibrio* spp. was demonstrated as a cleared zone around bacterial colonies. As expected from the previous reports (12, 19, 20), sizable zones of clearance were observed in all *Vibrio* strains tested in this study (upper panels, Fig. 9A). Although 42°C is not a temperature that is lethal to the *Vibrio* strains tested in this study, all the species cultured at 42°C showed an almost complete loss of exoproteolytic activity (lower panels, Fig. 9A). Reduced exoproteolytic activity was not due to a loss of enzymatic activity at high temperature, since the *V. vulnificus* exoproteases predominantly composed of the elastase VvpE showed similar levels of enzymatic activity at 30°C and 42°C (Fig. S4). Thus, it is presumed that exoprotease expression was reduced at 42°C. To elucidate the reason for the reduction in or lack of exoprotease expression at a high temperature, the cellular contents of each QS master regulator were examined in each *Vibrio* spp. by using polyclonal antibodies specific to *V. cholerae* HapR, *V. parahaemolyticus* OpaR, or *V. harveyi* LuxR. As shown in SmcR in the 42°C-treated *V. vulnificus* experiment (Fig. 3B), the protein bands for each QS master regulator gradually disappeared as the cells were incubated at 42°C up to 2 h (Fig. 9B).

10.1128/mBio.02086-17.4 FIG S4  Azocasein assay for exoprotease activity. Activities of the *V. vulnificus* exoproteases were measured by monitoring the extent of azocasein degradation upon incubation with the spent medium of *V. vulnificus* cells grown at 30°C or 42°C. Azocasein solution (20 mg/ml) was mixed with an equal volume of the bacterial cell-free supernatants and incubated for 1 h at 30°C or 42°C. The azo dye released by proteolytic activity in the supernatant was determined by spectrometry at 440 nm (OD_440_). Download FIG S4, TIF file, 0.04 MB. Copyright © 2018 Lee et al. 2018 Lee et al. This content is distributed under the terms of the Creative Commons Attribution 4.0 International license.

To investigate the effect of heat shock proteolysis of the QS master regulators on biofilm formation in each of the *Vibrio* spp., the levels of biofilms formed at 30°C or 42°C were estimated (Fig. 9C). At 42°C, *V. parahaemolyticus* formed highly increased levels of biofilms on the surface area of the air-liquid interface as well as on the bottom of the assay tubes, similarly to the results observed for *V. vulnificus* (Fig. 1). *V. cholerae* produced increased biofilms on the air-liquid interface area of the tubes. In contrast, *V. harveyi* biofilms at the air-liquid interface area were reduced in level, but the levels of the biofilms formed at the bottom of the tubes were highly increased. These results demonstrated that the total levels of the biofilms formed at 42°C were higher than those seen at 30°C in all four *Vibrio* species, although the QS regulatory mechanisms for biofilm formation were not identical among the species of *Vibrio*.

### Proteolysis of the LuxR homologues by ClpPA and Lon.

*V. cholerae*, *V. parahaemolyticus*, and *V. harveyi* are presumed to have both ClpPA and Lon in the heat shock response, based on the *in silico* analyses of each ClpP (VC_1922 [*V. cholerae*]; VP0917 [*V. parahaemolyticus*]; and LA59_05535 [*V. harveyi*]), ClpA (VC_1144 [*V. cholerae*]; VP1014 [*V. parahaemolyticus*]; and LA59_06115 [*V. harveyi*]), and Lon (VC_1920 [*V. cholerae*]; VP0919 [*V. parahaemolyticus*]; and LA59_05545 [*V. harveyi*]). They were approximately 96% to 98%, 91% to 93%, and 90% to 94% identical to the amino acid residues of the *V. vulnificus* ClpP, ClpA, and Lon, respectively. To elucidate whether the three SmcR homologues are directly cleaved by ClpPA and/or Lon, as shown in *V. vulnificus* (Fig. 7), *in vitro* proteolysis reactions were performed using recombinant HapR, OpaR, and LuxR proteins and *V. vulnificus* ClpPA and Lon. Equal amounts of purified LuxR homologues and ClpA were mixed in reaction buffer, and the reaction was initiated by the addition of various concentrations of ClpP. After 60 min, the substrate proteins remaining in the reaction mixtures were fractionated by SDS-PAGE, and the results showed that all three SmcR homologues were cleaved by ClpPA with differential degrees of proteolysis (Fig. 9D). The 50% effective concentrations (EC_50_s) of ClpP (i.e., the concentrations that were effective with respect to cleaving 50% of HapR, OpaR, and LuxR) were about 0.27, 0.70, and 0.57 μM, respectively. Similarly, 60-min incubations of each SmcR homologue in the presence of various concentrations of Lon demonstrated that all of them were well cleaved by Lon (Fig. 9E), with EC_50_s ranging from 0.07 to 0.11 μM.

## DISCUSSION

The QS master regulators, such as LuxR in *V. harveyi*, HapR in *V. cholerae*, OpaR in *V. parahaemolyticus*, and SmcR in *V. vulnificus*, are distinct from LuxR of *V. fischeri* in terms of their protein structures, biochemical characteristics, and genetic organization. These QS master regulators, belonging to the TetR family of transcription factors, bind the regulatory regions of target genes as a dimeric form without associating with AI molecules. Instead, they sense the specific AIs via a series of signal transduction pathways involving membrane-bound AI receptors and phosphorelay components (9).

Mechanisms regulating the expression of the QS master regulator have been extensively studied (9). The LuxO transcription factor plays a major role in repressing the expression of the QS master regulators at the posttranscription level via the activity of small RNA (sRNA) and the RNA chaperone Hfq. At low cell densities, translation of *luxR*, *hapR*, and *smcR* is repressed by multiple sRNAs (named "Qrr"), transcription of which is induced by the phosphorylated form of LuxO (21, 22). In addition to the posttranscription regulation, the expression levels of *smcR*-homologous genes are regulated at the level of transcription. Transcription of *smcR* is repressed by LuxT at low cell densities, transcription of which is activated by LuxO (23). In *V. cholerae*, AphA is known to repress *hapR* expression (24, 25). Autoregulation of their transcription, in which the transcription of *hapR* and *luxR* is repressed by HapR and LuxR, respectively, has been previously reported (24). Transcription of *smcR* is also autoregulated by binding of SmcR to two sites in the upstream regions of *smcR* (M.-A. Lee and K.-H. Lee, unpublished data). In *V. alginolyticus*, LuxR binds one site near its promoter region for transcription repression or LuxR binds the other site in its upstream region for transcription activation (26, 27). In the present report, we present another mechanism regulating expression of QS master regulators at the posttranslation level via proteolytic activities of Lon and ClpP in at least the four *Vibrio* species tested in this study (Fig. 4, 7, and 9D and E).

ClpP and Lon are described as heat-inducible proteins in *Escherichia coli*, and both are transcribed by sigma H (28). Similarly, two proteases were also induced in *V. vulnificus* cells growing under high-temperature conditions, although the apparent cellular levels were detectable in the cells growing under the normal conditions (see Fig. S2 in the supplemental material). ClpP, an ATP-dependent serine protease, is important for protein quality control under various stress conditions (29). ClpP protease requires a cognate chaperone protein for substrate specificity such as ClpX or ClpA containing the ATPase domain. ClpPX has been well characterized in the specific proteolysis of RssB-bound sigma S complex in *E. coli* (30, 31). ClpA is able to unfold the stable protein structures, allowing ClpP to cleave its substrates (32). HemA, RepA, and SsrA-tagged proteins have been identified as ClpPA-specific substrates (33–35). Here, we added SmcR and the other QS master regulators (i.e., HapR, OpaR, and LuxR) to the list of proteins recognized by ClpA (Fig. 7A and B and 9D). The cellular level of ClpA increased by approximately 3-fold to 4-fold in *V. vulnificus* cells grown at 42°C compared to the level seen in cells grown at 30°C (Fig. S2), but *E. coli clpA* has not been reported to belong to the sigma H regulon, and its expression was not induced by heat shock (36, 37).

The dimeric SmcR protein is an active form binding to the SmcR-binding consensus sequences in *V. vulnificus* QS-regulated genes (18). Thermal unfolding curves derived from CD analysis of the recombinant SmcR proteins revealed that the monomeric form of SmcR with Tyr171 and Cys198 replaced with Ala showed less thermostability than the dimeric SmcR (Fig. 8C). Both ClpPA and Lon showed higher proteolytic activities with respect to the monomeric form of SmcR than with respect to the dimeric SmcR (Fig. 8D and E). These results suggest that monomeric SmcR, which is a transcriptionally inactive form, is more susceptible to proteolysis than dimeric SmcR. Association of *V. alginolyticus* LuxR with its binding site has been reported to become unstable at 42°C (27). If this is similar to what occurs with other *Vibrio* species, it is speculated that the transcriptionally inactive monomeric forms of the QS master regulators would be favorable substrates for ClpPA and Lon under high-temperature conditions, where the cellular levels of ClpP, ClpA, and Lon were increased as well (Fig. S2).

The *V. fischeri* QS system has also been documented to be controlled by heat shock. *V. fischeri luxI* transcription is repressed by LexA, whose expression is dependent on sigma H (38). In addition, *Pseudomonas aeruginosa* LasI, which is homologous to *V. fischeri* LuxI, is degraded by Lon, leading to reduced synthesis of AI and further negative regulation of its downstream QS system, RhlR/I (39). Thus, it is presumed that the QS systems do not fully operate in heat-treated cells, since the cellular levels of QS master regulators are reduced in *V. vulnificus*, *V. harveyi*, *V. cholerae*, and *V. parahaemolyticus* and the extracellular levels of AIs are reduced in *V. fischeri* and *P. aeruginosa*.

The marine bacterium *V. vulnificus* may experience a wide range of temperatures during its life cycle, which is composed of the planktonic style under conditions of seasonally fluctuating temperatures of seawater and the pathogenic style under conditions of the relatively higher temperature of its hosts. Upon entering the human gut, *V. vulnificus* would be exposed to intestinal temperatures, which usually range from 36.8 to 39.3°C but may reach above 40°C (17). *V. vulnificus* grows well at 42°C, although its maximum yield at that temperature was slightly less than that seen with growth at 30°C or 37°C (Fig. S1). In this study, it was observed that *V. vulnificus* produced extraordinarily increased biofilm sizes at higher incubation temperatures (Fig. 1). Formation of biofilms is considered a survival strategy for many bacteria facing a variety of stresses, and they respond to diverse stress conditions by increasing their levels of biofilm formation (40). Increased formation of mature biofilms requires induction of the biofilm maturation components or repression of the biofilm dispersion components. *V. vulnificus* entering high-temperature host environments should be exposed to various host defense mechanisms, and thus its survival might be achieved by inducing the transition of its growth state from plankton to biofilm. This transition is facilitated by turning off the production of biofilm dispersion components, such as CPS (Fig. 2), production of which is activated by QS. Many bacterial cells in biofilms are reported to be more resistant to diverse physicochemical parameters than planktonic cells. Thus, *V. vulnificus* cells in biofilms which have been formed in host environments and then excreted from hosts might show better survival in natural, nonhost environments as well.

Increased biofilm formation was not limited to *V. vulnificus* cells treated to 42°C. The heat shock also resulted in an increase in the total biofilm masses of cells of the other *Vibrio* spp. (Fig. 9C). Further studies with other *Vibrio* spp. showed that high temperature is able to deactivate QS regulatory circuits, as evidenced by the decreased production of exoprotease (Fig. 9A). The gradual disappearance of the cellular QS master regulators in each *Vibrio* species during a 2-h exposure to 42°C (Fig. 9B) was presumed to have been caused by proteolysis of ClpPA and Lon (Fig. 9D and E). However, the biofilms formed on the surfaces of the air-liquid interface or the bottom of the assay tubes were distinct in *V. cholerae* and *V. harveyi* cells treated at 42°C. At 42°C, *V. cholerae* biofilms were predominantly present at the air-liquid interface area of the tubes, but most *V. harveyi* biofilms were formed on the bottom of tubes (Fig. 9C). These results suggest that the QS regulatory mechanisms for biofilm formation are not identical among the species of *Vibrio*. In *V. cholerae*, HapR represses the production of biofilm maturation components, such as the exopolysaccharide Vps (15). It has been reported that *V. cholerae* growing at 15°C formed more biofilms on the bottom surface than *V. cholerae* growing at 37°C (41). The biofilm formation was mediated by increased levels of cyclic diguanylate (c-di-GMP) under conditions of lower incubation temperatures. It would be fruitful to study the interaction between two regulatory pathways mediated by QS and c-di-GMP with regard to the incubation temperature as well as the biofilm-forming location. Swimming motility derived from polar flagella plays a critical role in the initial attachment to surfaces and is also important in the dispersal of cells from the biofilm structure. Since it was found that QS activates flagellar expression in *V. harveyi* (16), *V. harveyi* was shown to produce tightly formed biofilms on the bottom surface of assay tubes. In the case of *V. parahaemolyticus*, it is currently unknown whether OpaR-induced CPS production is required for the maturation or dispersal of biofilms, but the pattern of increased biofilm levels in the borosilicate tubes at 42°C was similar to that seen with the *V. vulnificus* biofilms formed at 42°C.

At least four *Vibrio* species showed significantly reduced abilities to turn on QS regulation under the heat shock condition due to intense proteolysis of their QS master regulators. This type of QS deactivation occurring through heat shock responses can be achieved even if the concentrations of AI molecules reach the levels representing high cell densities. One example of QS deactivation at high temperatures is represented by biofilm formation. In addition, many virulence factors are known to be induced by QS. The pathogenic *Vibrio* species enter the human hosts via consumption of contaminated foods and then encounter the numerous microorganisms in gut environments. If the expression of virulence factors is not desirable upon entering host environments, heat shock-mediated QS deactivation prevents pathogens from inducing virulence even in the presence of high concentrations of AI-2 molecules produced by gut microbiota.

## MATERIALS AND METHODS

### Bacterial strains, culture media, and oligonucleotide primers.

Bacterial strains and plasmids used in this study are listed in Table S1 in the supplemental material. Various *Vibrio* strains were grown at 30°C in LBS medium (Luria-Bertani [LB] medium containing NaCl at a final concentration of 2.5%) or AB medium (300 mM NaCl, 50 mM MgSO_4_, 0.2% vitamin-free Casamino Acids, 10 mM potassium phosphate, 1 mM l-arginine, pH 7.5; 42) supplemented with 1.0% fumarate (AB-fumarate; 3). *V. vulnificus* strains carrying a derivative plasmid of broad-host-range vector pRK415 were grown at 30°C in medium supplemented with 3 μg/ml tetracycline. *E. coli* strains were grown at 37°C in LB medium supplemented with 100 μg/ml ampicillin, 20 μg/ml chloramphenicol, or 15 μg/ml tetracycline. The primers used for the construction of mutants and the cloning of recombinant proteins are listed in Table S2.

10.1128/mBio.02086-17.5 TABLE S1  Bacterial strains and plasmids used in this study. Download TABLE S1, DOCX file, 0.02 MB. Copyright © 2018 Lee et al. 2018 Lee et al. This content is distributed under the terms of the Creative Commons Attribution 4.0 International license.

10.1128/mBio.02086-17.6 TABLE S2  Primers used in this study. Download TABLE S2, DOCX file, 0.01 MB. Copyright © 2018 Lee et al. 2018 Lee et al. This content is distributed under the terms of the Creative Commons Attribution 4.0 International license.

### Construction of deletion mutants. (i) Construction of *lon* deletion mutant.

A 647-bp DNA fragment containing the *lon* upstream region was amplified from the genomic DNA of *V. vulnificus* MO6-24/O using two primers, lonA-upF and lonA-upR. The PCR product was then cloned into pBluescript SK II(+) to produce pSK*lonA*up. A 514-bp DNA fragment containing the downstream region of *lon* was made using primers lonA-downF and lonA-downR and was cloned into the corresponding sites of pSK*lonA*up to result in pSK*lonA*up/down. A 1,161-bp DNA fragment of pSK*lonA*up/down digested with SalI and ApaI was ligated into a suicide vector, pDM4, to generate pDM4-Δ*lon*. The *E. coli* SM10λ*pir* strain carrying pDM4-Δ*lon* was conjugated with *V. vulnificus* MO6-24/O, and the exconjugants were selected on thiosulfate citrate bile sucrose (TCBS) medium. Deletion of *lon* in the selected colony was further confirmed by PCR using primers lonA-upF and lonA-downR. For complementation of the Δ*lon* mutant, a 2,352-bp DNA fragment containing the *V. vulnificus lon* gene, which had been amplified using two primers, lonA-comF and lonA-comR, was cloned into broad-host-range plasmid pRK415. The resultant plasmid, pRK415-*lon*, was transformed into *E. coli* SM10λ*pir* and then transferred to Δ*lon V. vulnificus* by conjugation (43).

### (ii) Construction of *clpP* deletion mutant.

A 719-bp DNA fragment containing the upstream region of *clpP* was amplified using *clpP*-upF and clpP-upR. The PCR product was then cloned into pBluescript SKII(+) to produce pSK*clpP*up. A 566-bp DNA fragment containing the *clpP* downstream region was made using primers clpP-downF and clpP-downR and was cloned into the corresponding sites of pSK*clpP*up to produce pSK*clpP*up/down. A 1,285-bp DNA fragment of pSK*clpP*up/down digested with ApaI and SacI was ligated into pDM4 to generate pDM4-Δ*clpP*. This plasmid was transferred to *V. vulnificus* as described above. The deletion of *clpP* was confirmed by PCR using primers clpP-upF and clpP-downR. For complementation of the Δ*clpP* mutant, a 627-bp DNA fragment containing the intact *clpP* gene, which had been amplified using *clpP*-comF and *clpP*-comR, was cloned into pRK415. The pRK415-*clpP* resultant was transformed into *E. coli* SM10λ*pir* and then transferred to the Δ*clpP* mutant by conjugation.

### (iii) Construction of *clpA* and *clpX* deletion mutants.

As described above, pSK*clpA*up was produced by inserting a 619-bp *clpA* upstream DNA fragment amplified using primers clpA-upF and clpA-upR. Then, pSK*clpA*up/down was produced by inserting an 819-bp *clpA* downstream DNA fragment amplified using primers clpA-downF and clpA-downR. The kanamycin resistance (Km^r^) gene (*nptI*) in pUC4K (Pharmacia) was isolated using BamHI and ligated to BamHI-digested pSK*clpA*up/down to generate pSK*clpA*up/*nptI*/down. Its insertion DNA, digested with ApaI and SacI, was ligated into pDM4 to generate pDM4-Δ*clpA*Km^r^. The candidate Δ*clpA* mutant was confirmed by PCR using primers clpA-upF and clpA-downR. The complementation plasmid for *clpA* was produced by ligating pRK415 with a 2,277-bp DNA fragment containing the intact *clpA* gene, which had been amplified using primers clpA-comF and clpA-comR.

Similarly, pSK*clpX*up/*nptI*/down was produced using a 670-bp *clpX* upstream DNA fragment (amplified by primers clpX-upF and clpX-upR), a 1,618-bp *clpX* downstream DNA fragment (amplified by primers clpX-downF and clpx-downR), and a BamHI-digested *nptI* gene. The resulting Δ*clpX* mutant was confirmed by PCR using primers clpX-upF and clpX-downR.

### Cloning and purification of recombinant proteins.

The following primer sets were used to amplify 618-, 627-, 2,277-, 993-, and 2,370-bp DNA fragments containing the complete open reading frame of the *smcR*, *clpP*, *clpA*, *rpoS*, and *lon* genes, respectively: SmcRexp-F/SmcRexp-R; ClpPexp-F/ClpPexp-R; ClpAexp-F/ClpAexp-R; RpoSexp-F/RpoSexp-R; and lonA-comF/lon6XHR. The amplified DNA fragments of *smcR*, *clpP*, *clpA*, and *rpoS* were cloned into pQE30, and that of *lon* was cloned into pRK415. The recombinant SmcR, ClpP, ClpA, and RpoS proteins were overexpressed in *E. coli* JM109 grown in the LB medium containing 1 mM IPTG (isopropyl-β-d-thiogalactopyranoside), and recombinant Lon was overexpressed in *V. vulnificus* MO6-24/O grown in the LBS medium containing 1 mM IPTG. The recombinant proteins were purified using a Ni^+^-nitrilotriacetic acid affinity column. In case of rLon, it was further subjected to the desalting step by dialyzing purified protein in a buffer containing 50 mM Tris-HCl (pH 8.0), 10 mM MgCl_2_, and 10% glycerol (44).

### Preparation of polyclonal antibodies against HapR, OpaR, and LuxR.

To construct the overexpression plasmids for LuxR homologues, 651-bp *V. cholerae hapR*, 618-bp *V. harveyi luxR*, and 615-bp *V. parahaemolyticus opaR* DNA fragments were amplified by using the following primer sets: HapRexp-F/HapRexp-R; LuxRexp-F/LuxRexp-R; and OpaRexp-F/OpaRexp-R. The *luxR* and *opaR* DNA fragments were cloned into pQE30, and the *hapR* DNA fragment was cloned into pET28a. To obtain the HapR-, LuxR-, and OpaR-specific polyclonal antibodies, 6-week-old female Sprague-Dawley rats were immunized intraperitoneally with 50 μg of each recombinant protein. The animals were boosted twice at 2-week intervals with the same amount of proteins. Four days after the third immunization, blood samples of rats were pooled and used for further experiments as polyclonal antibodies against each recombinant protein. The animals received humane care in accordance with our institutional guidelines and the legal requirements (no. IACUSGU2015_03). The specificity of the resulting antibodies was confirmed by Western blot analysis of the *E. coli* extracts expressing each recombinant proteins.

### Site-directed mutagenesis of SmcR.

Two amino acids residues responsible for the formation of dimeric SmcR (18) were mutagenized using primers carrying the substituted nucleotides. To change Y171 of SmcR to A, two sets of primers, SmcRexp-FB/SmcRY171AR and SmcRY171AF/SmcRexp-RH, were utilized. Two PCR products were simultaneously used as template DNA to produce the mutagenized *smcR* DNA using primers SmcRexp-FB and SmcRexp-RH. The resultant mutagenized *smcR* DNA was digested with BamHI and HindIII and was ligated to BamHI/HindIII-digested pQE30 to produce pQE-SmcRY171A. Then, another amino acid of SmcR, C198, was further changed to A by using SmcRexp-FB/SmcRC198ARH to produce pQE-SmcRY171A/C198A. The mutagenized nucleotide sequences were confirmed by DNA sequencing. The resultant plasmid was mobilized into *E. coli* JM109 to overexpress SmcR_Y171A/C198A_. Purified SmcR_Y171A/C198A_ was subjected to CD analysis to monitor the occurrence of secondary structure changes due to site-directed mutagenesis in SmcR by scanning the original SmcR (SmcR_WT_) and SmcR_Y171A/C198A_ using a spectropolarimeter (Jasco; Model SJ-815), as previously described (45). Far-UV CD spectra were recorded between 200 and 250 nm at 20°C. To observe the thermal stability of protein, the melting curves of SmcR_WT_ and SmcR_Y171A/C198A_ were obtained by measuring ellipticity [θ] at 222 nm and 30 to 95°C at a rate that increased 2°C/min. Purified SmcR_WT_ and SmcR_Y171A/C198A_ were placed in the buffer containing 50 mM sodium phosphate (pH 7.5) and 300 mM NaCl. For measuring the far-UV CD spectra, 2.3 mg/ml of each protein was injected into a cuvette with a path length of 0.1 cm. For measuring thermal stability, 0.23 mg/ml of each protein and a cuvette with a path length of 0.5 cm were used.

### *In vitro* proteolytic activity assay.

Degradation assays for analysis of rSmcR and other LuxR homologues were performed in ClpAP reaction buffer (50 mM HEPES [pH 7.5], 100 mM NaCl, 20 mM MgCl_2_, 1 mM DTT, 2 mM ATP, 10% glycerol) at 37°C as described by Jennings et al. (46). One microgram of rSmcR and rClpA was included in 20-μl total reaction mixtures. The reactions were initiated by adding rClpP (0 to 0.5 μg), and the reaction mixtures were incubated at 37°C for various time courses. For analysis of the Lon proteolytic activity, the assay was performed in Lon reaction buffer (50 mM Tris-HCl [pH 8.0], 10 mM MgCl_2_, 1 mM DTT, 2 mM ATP, 10% glycerol; 44). The reactions were stopped by the addition of SDS loading buffer (100 mM Tris-HCl [pH 6.8], 4% SDS, 0.2% bromophenol blue, 20% glycerol, 200 mM DTT) with boiling, and the resulting reaction mixtures were subjected to SDS-PAGE.

### Western blot analysis of SmcR and other LuxR homologues.

Wild-type and mutant *V. vulnificus* strains were cultured at 30°C in AB-fumarate until an optical density at 595 nm (OD_595_) of 1.0 was reached. Other *Vibrio* strains were inoculated into LBS. The contents of the culture tubes were divided in two tubes that were incubated in a water bath at a temperature of 30°C or 42°C, and the cells were harvested at the various time points. The cell lysates were obtained by sonication in TBST buffer (25 mM Tris-HCl [pH 7.4], 137 mM NaCl, 3 mM KCl, 0.05% [vol/vol] Tween 20). The cellular levels of SmcR in *V. vulnificus* and of other LuxR homologues in *V. cholerae*, *V. parahaemolyticus*, and *V. harveyi* were observed by immunoblotting performed with the specific polyclonal antibodies. For a loading control, immunogenic lipoprotein A (IlpA), the cellular level of which is known to be insensitive to heat shock treatment in *V. vulnificus* (K.-J. Lee and K.-H. Lee, unpublished), was also monitored in *Vibrio* lysates. The cellular levels of IlpA in the same lysates were examined using anti-IlpA polyclonal antibodies (47).

### Measurement of endogenous degradation of SmcR in *V. vulnificus.*

Antibiotic chase assays were performed as previously described (48). Freshly grown cells of wild-type, Δ*clpP*, and Δ*lon V. vulnificus* strains were concentrated and inoculated into prewarmed AB-fumarate medium (30°C or 42°C) containing 6 μg/ml tetracycline. At various incubation times (45 to 90 min) in a 30°C or 42°C water bath, aliquots were sampled and immediately frozen in liquid nitrogen. Wild-type cells maintained at 42°C were sampled at 0, 2.5, 5, 10, 20, 30, and 45 min, and the wild-type cells maintained at 30°C and the other cells were sampled at 0, 10, 20, 30, 45, 60, and 90 min. The cell lysates were prepared by thawing and sonication, and the remaining levels of SmcR in *V. vulnificus* cells were observed by immunoblotting analysis as described above. To estimate the half-life of SmcR in each strain, the intensities of SmcR were normalized to the SmcR intensity at time 0 and then plotted against incubation time.

### Biofilm formation assays.

Biofilms formed by *V. vulnificus* were observed in borosilicate tubes containing AB-fumarate medium at 30°C or 42°C without agitation. At the end of the incubation, the planktonic cell density in each biofilm tube was measured by spectrometric reading at 595 nm. To estimate the degrees of biofilm formation, biofilms were stained with 1.0% crystal violet after the medium and the planktonic cells in the vessels had been removed and the remaining biofilms were washed with PBS (137 mM NaCl, 2.7 mM KCl, 10 mM Na_2_HPO_4_, and 2 mM KH_2_PO_4_; pH 7.4; 49). The crystal violet-stained biofilms were eluted in 100% ethanol, and the amounts of dye were quantified by spectrometric reading at 550 nm (50). To examine the dispersal of cells in the biofilms, biofilms were formed in the borosilicate tubes containing 2 ml of AB-fumarate at 30°C for 48 h, and the culture supernatants were then discarded. The remaining biofilms were washed with PBS, resuspended in 4 ml PBS, and incubated at 30°C or 42°C for another 24 h to allow the second biofilms to be formed by the cells dispersed from the first biofilms (51). Then, the first and second biofilms around the air-liquid interfaces, which had been produced in 2 ml AB-fumarate broth and in 4 ml of PBS, respectively, were estimated by staining with crystal violet as described above.

### Analysis of CPS extracts.

*V. vulnificus* colonies grown for 48 h on the AB-fumarate agar plates were suspended in 10 ml of PBS to give a final cell density (OD_595_) of 100, and their CPS proteins were prepared from bacterial cell pellets after loosely associated exopolysaccharides (EPS) had been washed away, as previously described (6). Resultant CPS extracts were run on a 5% stacking gel and stained with Stains-All (Sigma), as described by Enos-Berlage et al. (52). The galacturonic acid content in the CPS extracts was determined by a colorimetric assay using d-(+)-galacturonic acid monohydrate (Sigma) as a standard, as previously described (53). The estimated carbohydrate concentrations were expressed as nanograms of galacturonic acid equivalents in 1 μl of extract.

### Quantitative RT-PCR.

Wild-type *V. vulnificus* cells were cultured at 30°C in AB-fumarate until an OD_595_ of 1.0 was reached and then were divided and placed into two tubes. Each tube was incubated at 30°C or 42°C. Reverse transcription reactions were performed with the total RNA isolated from *V. vulnificus*, which had been harvested at several incubation time points, using Moloney murine leukemia virus (M-MLV) reverse transcriptase and a random 6-mer primer (TaKaRa). The cDNA was analyzed by quantitative RT-PCR using a LightCycler 480 II system (Roche). The transcripts of the CPS gene cluster were normalized to the *gap* transcripts in each sample. Absence of genomic DNA contamination in the cDNA preparation was confirmed by omitting the addition of M-MLV reverse transcriptase in the reaction mixture for quantitative RT-PCR. Threshold cycle (*C*_*T*_) values representing the relative quantities of the target and reference gene transcripts in each sample were obtained and calculated by the 2^−ΔΔCT^ method (54).

### Luminescence assay for measuring promoter activity of the CPS gene cluster.

To monitor the expression of the CPS cluster in wild-type and mutant strains of *V. vulnificus*, promoterless *luxAB* plasmid pCB014 containing a DNA fragment ranging from position −326 to position +439 relative to the transcription initiation site for the CPS gene cluster (3) was used. *V. vulnificus* cells harboring this *luxAB*-transcription fusion reporter were cultured at 30°C in AB-fumarate broth supplemented with 3 μg/ml tetracycline. The amounts of cell masses were then determined by measuring OD_595_. Levels of light production by cells in the presence of 0.06% (vol/vol) *n*-decyl aldehyde were determined by using a luminometer (TD-20/20 luminometer; Turner Designs). Specific bioluminescence was calculated by normalizing the relative light units (RLU) with respect to cell mass (OD_595_).

### Statistical analyses.

Results were expressed as means ± standard deviations of data from at least three independent experiments. Statistical analysis was performed using Student’s *t* test (Systat Program, SigmaPlot version 9; Systat Software, Inc.). *P* values are presented in the corresponding figures or are indicated in the figures by one asterisk (*) or two asterisks (**) (0.05 < *P* < 0.001 or *P* < 0.001, respectively).
