# Supplementary figures and images for: Role of Heat Shock Proteases in Quorum-Sensing-Mediated Regulation of Biofilm Formation by Vibrio Species
Source: mBio. 2018 Jan 2;9(1):e02086-17. doi: 10.1128/mBio.02086-17 (PMC5750401; doi:10.1128/mBio.02086-17)

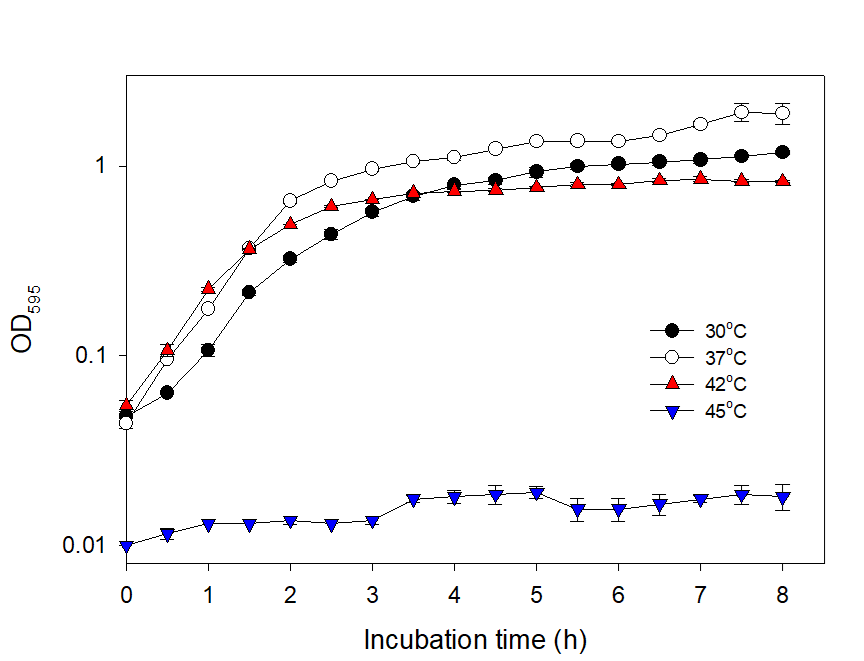

Supplement: FIG S1 [file mbo001183661sf1.tif]

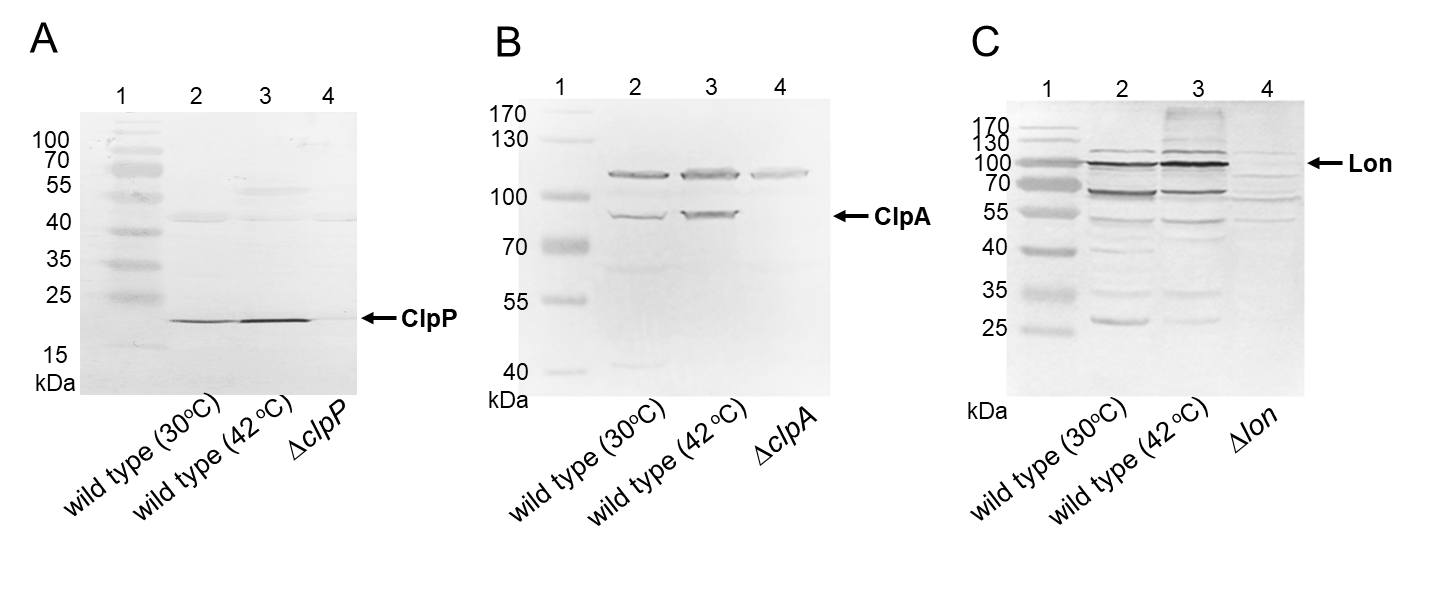

Supplement: FIG S2 [file mbo001183661sf2.tif]

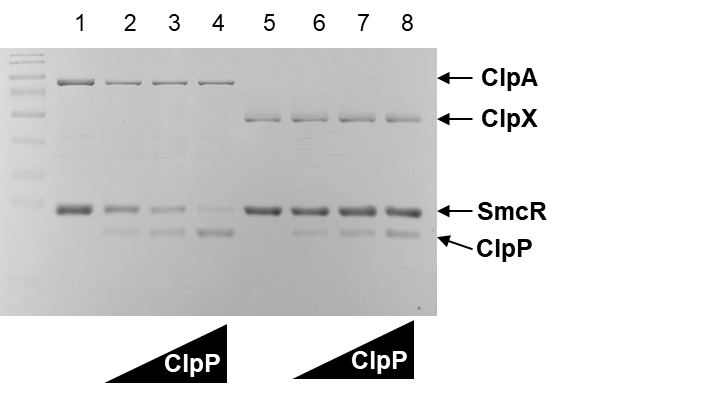

Supplement: FIG S3 [file mbo001183661sf3.tif]

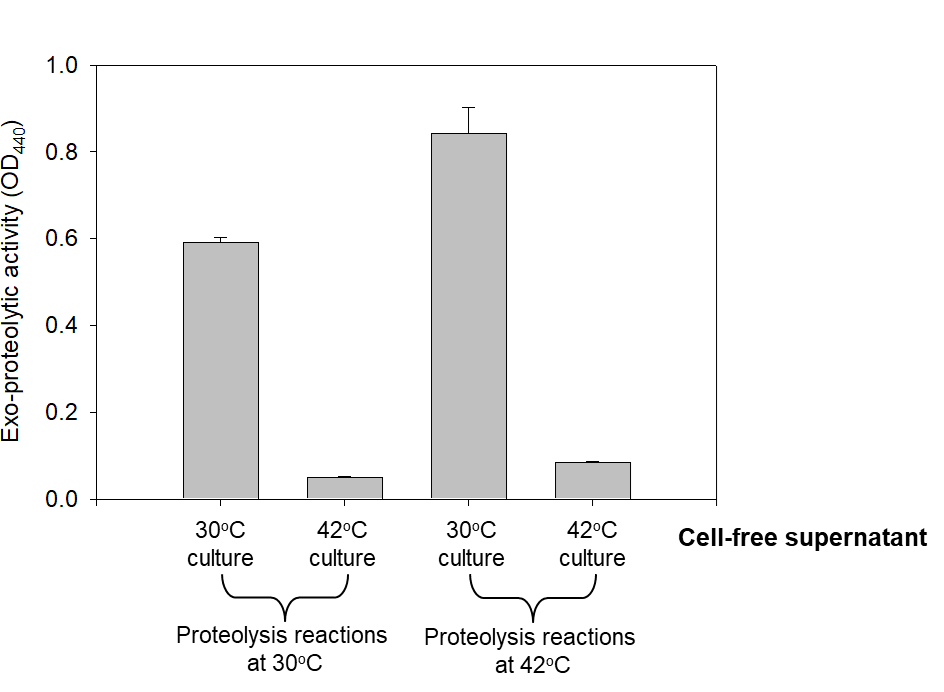

Supplement: FIG S4 [file mbo001183661sf4.tif]
