# Supplementary material for: Role of Heat Shock Proteases in Quorum-Sensing-Mediated Regulation of Biofilm Formation by Vibrio Species
Source: mBio. 2018 Jan 2;9(1):e02086-17. doi: 10.1128/mBio.02086-17 (PMC5750401; doi:10.1128/mBio.02086-17)
Supplement: TABLE S1 [file mbo001183661st1.docx]

**Table S1. Bacterial strains and plasmids used in this study**

| Strain/plasmid | Genotype | Source/reference |
| --- | --- | --- |
| ***E. coli*** |  |  |
| DH5α | (Φ80 *lacZ*Δ*M15*) *recA1 endA1 gyrA96 relA1 thi-1 hsdR17*(r_K_^–^m_K_^–^) *supE44 deoR*Δ (*lacZYA-argF*)*U169* | Laboratory collection |
| SM10*λpir* | *thi-1 thr leu tonA lacY supE recA*::  Rp4-2-Tc::Mu*λpir*, OriT of RP4, Km^R^ | 1 |
| JM109  BL21 (DE) | *endA1 recA1 gyrA96 thi-1 hsdR17*(r_K_^–^m_K_^–^) *relA1supE44* Δ(*lac-proAB*)[F’*traD36proABlacI*^q^ZΔ*M*15]  *E. coli* strain B F^-^ *dcm ompT hsdS* (r_B_^-^ m_B_^-^) *galλ* (DE3) | Qiagen  Invitrogen |
| ***V. vulnificus*** | |  |
| MO6-24/O | Clinical isolate | 2 |
| CB504 | MO6-24/O, *wbpP*::mini-*Tn*5 lacZ1-Km*^R^* | 3 |
| HS031  KL321 | MO6-24/O, *smcR*, Km^R^  MO6-24/O, Δ*clpP* | 4  This study |
| KL322  KL421  SM801  ***V. cholerae***  ATCC14033  ***V. harveyi***  B392  ***V. parahaemolyticus***  RIMD2210633  **Plasmids** | MO6-24/O, Δ*clpA*, Km^R^  MO6-24/O, Δ*lon*, Km^R^  MO6-24/O, Δ*clpX*, Km^R^  Serovar O1, biotype ElTor, serotype Inaba  unknown  Serotype O3:K6 | This study  This study  This study  ATCC  5  ATCC/BAA-238 |
| pRK415  pRK415-*clpP*  pRK415-*clpA*  pRK415-*lon*  pRK415-*lon*6XHis  pBluescript II SK(+)  pDM4  pUC4K  pSK*clpX*up  pSK*clpX*up/down  pSK*clpX*up/*nptI*/down  pSM801  pSK*clpP*up  pSK*clpP*up/down  pDM4-Δ*clpP*  pSK*clpA*up  pSK*clpA*up/down  pSK*clpA*up/nptI/down  pDM4-Δ*clpA*Km^r^ | IncP *ori,* broad-host-range vector; *oriT* of RP4; Tc^R^  pRK415 containing *V. vulnificus clpP* gene*,* Tc^R^  pRK415 containing *V. vulnificus clpA* gene*,* Tc^R^  pRK415 containing *V. vulnificus lon* gene*,* Tc^R^  pRK415 containing *V. vulnificus lon* gene with 6XHis codons*,* Tc^R^  Cloning vector, Ap^r^  Suicide vector, *ori*R6K, Cm^r^  *nptI*, Ap^r^, Km^r^  pBluescript II SK(+) with 670-bp *V. vulnificus* *clpX* upstream region  pSK*clpX*up with 1618-bp *V. vulnificus* *clpX* downstream region  pSK*clpX*up/down with 1.2-Kb *nptI* gene  pDM4 with 3.6-Kb DNA of internally deleted and *nptI* inserted *clpX*  pBluescript II SK(+) with 566-bp *V. vulnificus* *clpP* upstream region  pSK*clpP*up with 719-bp *V. vulnificus* *clpP* downstream region  pDM4 with 1.3-Kb DNA of internally deleted *clpP*  pBluescript II SK(+) with 819-bp *V. vulnificus* *clpA* upstream region  pSK*clpA*up with 819-bp *V. vulnificus* *clpA* downstream region  pSK*clpA*up/down with 1.2-Kb *nptI* gene  pDM4 with 2.7-Kb DNA of internally deleted and *nptI* inserted *clpA* | 6  This study  This study  This study  This study  Stratagene  7  Pharmacia Biotech  This study  This study  This study  This study  This study  This study  This study  This study  This study  This study  This study |
| pSK*lonA*up  pSK*lonA*up/down  pDM4-Δ*lon*  pCB014 | pBluescript II SK(+) with 647-bp *V. vulnificus* *lon* upstream region  pSK*lonA*up with 514-bp *V. vulnificus* *lon* downstream region  pDM4 with 1.2-Kb DNA of internally deleted *lon*  pHK0011containing the upstream region of the CPS-cluster (*wza-luxAB*);Tc*^R^* | This study  This study  This study  3 |
| pQE30  pQE-SmcR  pQE-SmcR_Y171A/C198A_  pQE-ClpP  pQE-ClpA  pQE-RpoS | Expression vector, N-terminal 6-His tag, Ap^R^  pQE30 containing the *V. vulnificus smcR* ORF, Ap^R^  pQE30 containing *smcR* Y171A and C198A mutated region, Ap^R^  pQE30 containing the *V. vulnificus clpP* ORF, Ap^R^  pQE30 containing the *V. vulnificus clpA* ORF, Ap^R^  pQE30 containing the *V. vulnificus rpoS* ORF, Ap^R^ | Qiagen  This study  This study  This study  This study  This study |
| pQE-VhLuxR  pQE-VpOpaR  pET28a  pET28a-VcHapR | pQE30 containing the *V. harveyi luxR* ORF, Ap^R^  pQE30 containing the *V. parahaemolyticus opaR* ORF, Ap^R^  Expression vector; T7 *lac* promoter, *oriF1*; Km^R^  pET28a containing the *V. cholerae hapR* ORF, Km^R^ | This study  This study  Novagen  This study |

REFERENCES

1. Simon R, Priefer U, Pühler A. 1983. A broad host range mobilization system for in vivo genetic engineering: transposon mutagenesis in gram negative bacteria. Nat Biotechnol **1**:84-791.

2. Wright AC, Simpson LM, Oliver JD, Morris JG Jr. 1990. Phenotypic evaluation of acapsular transposon mutants of *Vibrio vulnificus*. Infect Immun **58**:1769-1773.

3. Kim HS, Park SJ, Lee KH. 2009. Role of NtrC-regulated exopolysaccharides in the biofilm formation and pathogenic interaction of *Vibrio vulnificus*. Mol Microbiol **74**:436-453.

4. Jeong HS, Lee MH, Lee KH, Park SJ, Choi SH. 2003. SmcR and cyclic AMP receptor protein coactivate *Vibrio vulnificus* *vvpE* encoding elastase through the RpoS-dependent promoter in a synergistic manner*.* J Biol Chem **278**:45072-45081.

5. Reichelt JL, Baumann P. 1973. Taxonomy of the marine, luminous bacteria. Arch Mikrobiol **94**:283-330.

6. Keen NT, Tamaki S, Kobayashi D, Trollinger D. 1988. Improved broad-host-range plasmids for DNA cloning in Gram-negative bacteria. Gene **70**:191–197.

7. Milton DL, O’Toole R, Hörstedt P, Wolf-Watz H. 1996. Flagellin A is essential for the virulence of *Vibrio anguillarum*. J Bacteriol **178**:1310–1319.
