# Supplementary material for: Role of Heat Shock Proteases in Quorum-Sensing-Mediated Regulation of Biofilm Formation by Vibrio Species
Source: mBio. 2018 Jan 2;9(1):e02086-17. doi: 10.1128/mBio.02086-17 (PMC5750401; doi:10.1128/mBio.02086-17)
Supplement: TABLE S2 [file mbo001183661st2.docx]

**Table S2. Primers used in this study**

| Primer name | Sequences (5’🡪3’) |
| --- | --- |
| lonA-upF  lonA-upR  lonA-downF  lonA- downR  lonA-comF  lonA-comR  clpP-upF  clpP-upR  clpP-downF  clpP-downR  clpP-comF  clpP-comR  clpA-upF  clpA-upR  clpA-downF  clpA-downR  clpA-comF  clpA-comR  clpX-upF  clpX-upR  clpX-do wnF  clpx-dow nR  SmcRexp-F  SmcRexp-R  ClpPexp-F  ClpPexp-R  ClpAexp-F  ClpAexp-R  RpoSexp-F  RpoSexp-R  lonA-comF  lon6XHR  HapRexp-F  HapRexp-R  LuxRexp-F  LuxRexp-R  OpaRexp-F  OpaRexp-R  SmcRY171AR  SmcRY171AF  SmcRC198ARH | GATCGTCGACGGAACGTTCCGAGCGTATCGAGATCCC  GATCCTCGAGGTTTCTTGACGCGGTTACGGATGCGC  GATCCTCGAGGGCTGCACATCGTGGTGGTATTACAAC  GATCGGGCCCCCAACTAGAGCTACTTGATCACCAGAC  CCCAAGCTTATGAACTTGGAACGTTCCGAG  CGGGATCCCTATTTTTTAGCGTCAAACTCCACCCC  GATCGGGCCCGTTGAAGCACGTCAACTACCAGAACTAAACG  ­CGGGATCCGGCGACATTGCATTTTTTTCTTGGTAGCTCAT  CGGGATCCGACTCATCGCAGTGCATAATTTGCCTGCGC  GATCGAGCTCATCTTCGCCCACATAACCCGCTTCTGTTAG  GATCAAGCTTATGAGCTACCAAGAAAAAAATGCAATG  ­GATCGGATCCTTATGCACTGCGATGAGTCAATACTG  ­GATCGGGCCCGCAGCCTGCTGTGGCGTGAATTAG  GATCGGATCCCCAACAATGCGAGTAGGAGGTGTTC  GATCGGATCCCGCGTGAAGAAGCGGTTCATTAAAAG  GATCGAGCTCCATCGTAACGGAACCATGTATCCCCC  GATCCTGCAGATGCTTAATAAAGAATTAGAGTCGAG  GATCGGATCCCTTAATGAACCGCTTCTTCACGCG  CGAGCTCACCTAACTCACGTGTGATGATTCACCAGCC  ­CGGGATCCAGCAAGATGTTACTCTTACCTAGCTCCACG  CGGGATCCCGTGTTGCTAGAAACCATGTATGAACTGCC  GCGGGCCCTCATTGAACGAGTTGTTCTGTTCTGGGTCC  CGGGATCCATGGACTCAATCGCAAAGAGA  GATCAAGCTTCTGGTGTTATTCGTGCTCGCG  GATCGAATTCATTAAAGAGGAGAAATTAACTATGAGCTACCAAGAAAAAAATGCAATG  GATCGGATCCCTAGTGATGGTGATGGTGATGTGCACTGCGATGAGTCAATACTGCG  GATCGAATTCATTAAAGAGGAGAAATTAACTATGCTTAATAAAGAATTAGAGTCGAGC  GATCGGATCCCTAGTGATGGTGATGGTGATGATGAACCGCTTCTTCACGCGTTGTC  GATCGGATCCTGAAAAGAGGAGAAATTAACTATGCATCACCATCACCATCACATGAGTATCAGCAACACAGTCACC  GATCAAGCTTTCAATCCATATCGATATCAAACAAATTTTCC  CCCAAGCTTATGAACTTGGAACGTTCCGAG  CGGGATCCCTAGTGGTGGTGGTGGTGGTGTTTTTTAGCGTCAAACTCCAC  GATCGGATCCATGCTCAATCAACAACTCAATTG  GATCAAGCTTCTAGTTCTTATAGATACACAGC  GATCGGATCCATGGACTCAATTGCAAAGAGACC  GATCAAGCTTAGTGATGTTCACGGTTGTAGATG  GATCGGATCCATGGACTCAATTGCAAAGAGACC  GATCAAGCTTAGTGTTCGCGATTGTAGATGC  CAAACAGCGAGGCACAAATGCCGTGG  CCACGGCATTTGTGCCTCGCTGTTT  GATCAAGCTTC*TTA*TTCGTGCTCGCGTTTATAGATGGCTAGCATGTC |
